# Supplementary material for: Perception of the use of a telephone interpreting service during primary care consultations: A qualitative study with allophone migrants
Source: PLoS One. 2022 Mar 15;17(3):e0264832. doi: 10.1371/journal.pone.0264832 (PMC8923434; doi:10.1371/journal.pone.0264832)
Supplement: S1 Appendix — (DOCX) [file pone.0264832.s001.docx]

**Supplemental Appendix 1**

**S1 Appendix. Interview guide.**

1. Can you tell me about how you came to France? (How long have you been here? Context? Reasons? What country are you from? Family situation ? Age ?)
2. I understand that you saw the doctor, and had help from the interpreting service. Can you tell me about that?

Did you know before the consultation that you could have an interpreter?

What was your reaction when you found out?

During the consultation, did the interpreter participate right from the beginning?

3- Can you tell me what contribution the interpreter’s participation made?

4- Did the interpreter’s participation change anything in your relation with the doctor ?

5- Can you describe how you felt about the presence of this stranger (i.e. the interpreter)?

6- Is it important to you that the interpreter be the same sex as you? Why?

7- How do you rate the interpreter’s translation? Was the language chosen for the translation appropriate? Did you understand the interpreter well?

8- How did you feel about the fact that the interpreter wasn’t present on site?

9- In your opinion, how useful is this type of consultation?

10- Would you like to have an interpreter again during your future appointments?

11- In your opinion, what could be improved?

12- Do you have anything else to say about things that we didn’t cover?
