# Supplementary material for: Perception of the use of a telephone interpreting service during primary care consultations: A qualitative study with allophone migrants
Source: PLoS One. 2022 Mar 15;17(3):e0264832. doi: 10.1371/journal.pone.0264832 (PMC8923434; doi:10.1371/journal.pone.0264832)
Supplement: S1 Table — (DOCX) [file pone.0264832.s002.docx]

**S2 Table. Consolidated criteria for reporting qualitative research (COREQ) [1].**

| **N° Item** | **Guide questions/ description** | **Reported on** |
| --- | --- | --- |
| **Domain 1: Research team and reflexivity** | | |
| Personal characteristics | | |
| 1. Interviewer/facilitator | Which author/s conducted the interview or focus group? | Each allophone migrant participated in a single semi-structured interview with a researcher (MJ: MD, GP, Primary Care researcher, female). |
| 2. Credentials | What were the researcher’s credentials? E.g. PhD, MD |  |
| 3.Occupation | What was their occupation at the time of the study? |  |
| 4. Gender | Was the researcher male or female? |  |
| 5. Experience and training | What experience or training did the researcher have? |  |
| Relationship with participants | | |
| 6. Relationship established | Was a relationship established prior to study commencement? | Eligible patients (n=32) were first identified by the GP at the primary care group practice in Bar-sur-Seine, and were then contacted by the social worker in the accommodation centre. |
| 7. Participant knowledge of the interviewer | What did the participants know about the researcher? | Each allophone migrant participated in a single semi-structured interview with a researcher (MJ: MD, GP, Primary Care researcher, female). |
| 8. Interviewer characteristics | What characteristics were reported about the interviewer/facilitator? e.g. Bias, assumptions, reasons and interests in the research topic |  |
| **Domain 2: Study design** | | |
| Theoretical framework | | |
| 9. Methodological orientation and Theory | What methodological orientation was stated to underpin the study? e.g. grounded theory, discourse analysis, ethnography, phenomenology, content analysis | The data from the interviews were analysed using thematic analysis. |
| Participant selection | | |
| 10. Sampling | How were participants selected? e.g. purposive, convenience, consecutive, snowball | Allophone migrant patients who had at least one consultation in primary care using the TIS were eligible. Eligible patients (n=32) were first identified by the GP at the primary care group practice in Bar-sur-Seine, and were then contacted by the social worker in the accommodation centre.  To account for the wide heterogeneity of allophone migrants likely to use the TIS, no inclusion criteria regarding age, sex, mother tongue, country of origin, socio-economic status or date of arrival in France were applied. |
| 11. Method of approach | How were participants approached? e.g. face-to-face, telephone, mail, email |  |
| 12. Sample Size | How many participants were in the study? | A total of 32 people were identified and contacted to participate. Thirteen of these agreed to participate, but 3 of them failed to show up at the agreed time for the interview. |
| 13. Non-participation | How many people refused to participate or dropped out? Reasons? |  |
| Setting | | |
| 14. Setting of data collection | Where was the data collected? e.g. home, clinic, workplace | The interviews were held in a dedicated room at the accommodation centre, which was known to all participants, and was quiet and informal. |
| 15. Presence of non-participants | Was anyone else present besides the participants and researchers? | Each allophone migrant participated in a single semi-structured interview with a researcher (MJ: MD, GP, Primary Care researcher, female). |
| 16. Description of sample | What are the important characteristics of the sample? e.g. demographic data, date | Table 1 |
| Data collection | | |
| 17. Interview guide | Were questions, prompts, guides provided by the authors? Was it pilot tested? | After discussing the circumstances of the participant’s arrival in France, the interview focused on the participant’s feedback concerning the use of the TIS during their medical consultation, and the impact of this service on the doctor-patient relationship. The main topics addressed were: how the patient came to France; how they learned about interpreting services; how they felt about the service, its usefulness and its impact on their healthcare experience. The full details of the interview guide are provided in the S1 Appendix. |
| 18. Repeat interviews | Were repeat interviews carried out? If yes, how many? | Each allophone migrant participated in a single semi-structured interview |
| 19. Audio/ visual recording | Did the research use audio or visual recording to collect the data? | With the oral consent of all the participants, the interviews were fully recorded. The recorder was placed in clear view on the table. |
| 20. Field notes | Were ﬁeld notes made during and/or after the interview or focus group? | No |
| 21. Duration | What was the duration of the interviews or focus group? | The average duration of the interviews was 34 minutes (range 23 to 63 minutes). |
| 22. Data saturation | Was data saturation discussed? | Interviews were performed until data saturation was reached (i.e. the point beyond which further interviews provide no new information) [24]. Data saturation was reached after nine interviews. |
| 23. Transcript returned | Were transcripts returned to participants for comment and/or correction? | No formal feedback was organized because the migrants only stay a few months in the CADA. Most of the asylum seekers interviewed were no longer present at the time the results became available. |
| **Domain 3: analysis and findings** | | |
| Data analysis | | |
| 24. Number of data coders | How many data coders coded the data? | Two rounds of analysis were performed independently by each of two researchers (MJ (female), LH (female)). The first round of analysis used open coding to identify the different themes present in the interviews, while the second round of analysis made it possible to classify these themes into major and minor themes, and to identify the relationships and hierarchies between them. |
| 25. Description of the coding three | Did authors provide a description of the coding tree? | As shown in Table 2, these clusters were assembled to generate the following three major themes: (i) multi-level difficulties for allophone migrants in the healthcare pathway; (ii) the interpreter as the cornerstone of the doctor-patient relationship; and (iii) limitations of the TIS. |
| 26. Derivation of themes | Were themes identiﬁed in advance or derived from the data? |  |
| 27. Software | What software, if applicable, was used to manage the data? | N/A |
| 28. Participant checking | Did participants provide feedback on the ﬁndings? | No formal feedback was organized because the migrants only stay a few months in the CADA. Most of the asylum seekers interviewed were no longer present at the time the results became available. |
| Reporting | | |
| 29. Quotation presented | Were participant quotations presented to illustrate the themes / ﬁndings? Was each quotation identiﬁed? e.g. participant number | YES |
| 30. Data and findings consistent | Was there consistency between the data presented and the ﬁndings? | YES |
| 31.Clarity of major themes | Were major themes clearly presented in the ﬁndings? | YES |
| 32. Clarity of minor themes | Is there a description of diverse cases or discussion of minor themes? | YES |

[1] Tong A, Sainsbury P, Craig J. Consolidated criteria for reporting qualitative research (COREQ): a 32-item checklist for interviews and focus groups. *Int J Qual Health Care* 2007; 19: 349–357.
