# Supplementary material for: Perception of the use of a telephone interpreting service during primary care consultations: A qualitative study with allophone migrants
Source: PLoS One. 2022 Mar 15;17(3):e0264832. doi: 10.1371/journal.pone.0264832 (PMC8923434; doi:10.1371/journal.pone.0264832)
Supplement: Attachment — Submitted filename: Response to Reviewers.docx [file pone.0264832.s003.docx]

**PONE-D-21-18663**

Revisions for submission: PONE-D-21-18663 ‘*Perception of the use of a telephone interpreting service during primary care consultations: A qualitative study with allophone migrants’*

Thank you very much for your feedback on our work. Please find our responses to the reviewers’ comments in the following table.

| **Review 11142021 – Major Review** | |
| --- | --- |
| **Reviewers Comment** | **Authors Response** |
| **Reviewer 1** | |
| **Thank you very much for a very interesting piece of research.** | Thank you very much for reviewing our work and for your enthusiasm. |
| **I miss a description of the themes heading each of the corresponding sections.** | Further to the suggestions from other Reviewers to re-structure the results section, the themes heading each of the sections have been renamed to be more descriptive, and the corresponding paragraphs describe what is covered by each heading. |
| **Further references about video interpreting in healthcare settins are the contributions by de Boe and by Krystallidou in Saalets, Heidi & Gert Brône (eds.) 2020. Linking up with video. Perspectives on interpreting practice and research. Amsterdam: John Benjamins.** | Thank you for the discovery of this very interesting publication. Krystallidou’s work was particularly relevant to our work, and we have added it in the discussion session. |
| **Reviewer 2** | |
| The research paper, number PONE-D-21-18663, falls into the field of health interpreting and deals with a question of interest, yet little explored, that of patients' perception of telephone interpreting. However, the manuscript requires a stronger background to justify the research. | Thank you very much for reviewing our work and for the constructive suggestions for improvement.  We hope our changes will be to your satisfaction. |
| The introduction is indeed rather weak, as it lacks depth and articulation. In the first paragraph, the authors briefly address language barriers and their implications, the need for an interpreter and the two broad categories of interpreter (informal vs. professional). They directly present in the second (and last) paragraph the context of TIS in France and the contextual elements that led to the research. There is no transition in between. The different interpreting techniques and their benefits are missing (see Wang & Fang (2019) for a comparison between telephone interpreting and in-person interpreting in terms of translation quality), as well as the scientific data on TIS. There are at least two systematic review available (Downes, Mervin, Byrnes & Scuffham, 2017; Joseph, Garruba, & Melder, 2018). Lee (2007) and Wang (2018) have also investigated in the perception of interpreters. In addition, the last sentence of the first paragraph needs clarification. | Thank you very much for bringing these relevant publications to our notice.  We have expanded the introduction to make a smoother transition between the ideas, and we have introduced the relevant references suggested by the Reviewer. |
| What do the authors mean by "qualitative terms" and "economic terms"? | Sorry for the misunderstanding, this has been rephrased. |
| The manuscript would gain in clarity, by reorganizing the structure and content of the Materials and methods section. The authors provided details regarding the population throughout the Method and at the beginning of Result section, while they should all be indicated in the same sub-section. | Thank you for this suggestion. However, as this was a prospective study, we decided to follow the conventional IMRAD structure. Accordingly, the description of how the population was identified is given in the methods, but the number of participants actually recruited is a result, and as such, is displayed in the results section. Therefore, we did not modify this section and count on your understanding. |
| The authors do not present the general topics explored in the interview (referring directly to the Supplementary Material is not sufficient). | Thank you for pointing this out. The main general topics explored during the interviews are now presented in the Data Collection section. |
| The sub-section “Telephone interpreting service (TIS)” does not appear necessary. | We agree, and we have deleted this part. |
| Funding information are already given at the end of the manuscript and details about ISM can directly be included in the introduction. Please group information and be more straightforward. | We have deleted the information within the manuscript and just left the funding section at the end of the manuscript. |
| Results are presented as a series of quotes, which does not do justice to the work involved by a qualitative analysis. | The Reviewer is correct in pointing out that a list of quotes is “reductive” and not an accurate reflection of the qualitative analysis. We have restructured the Results section by adding a new table (Table 2) presenting a summary of the overall analysis. |
| The discussion, in its present state, does not bring anything new. The literature has already underlined the implication language barriers can have in primary care, and in mental health. Bauer & Alegria (2010) have for example conducted a literature review on interpreting in mental health  . | We have revised the discussion section based on the publications suggested by the Reviewer.  We hope this new version will better highlight remaining challenges, and the lack of data available for TIS in the context of primary care. |
| Language polish is also strongly recommended. There are a number of linguistic typos throughout the manuscript. In the abstract, for example, the setting is presented as being the population, and not the accommodation center. I do not understand the label of the first theme "multiple translations of the language barrier". Can language barriers be translated? | The paper has been thoroughly revised by a native English-speaking medical writer with expertise in qualitative research. We remain open to any suggestions for further corrections.  Regarding "multiple translations of the language barrier", this has been rephrased. We hope that it will now be clearer. |
| Regarding "Ethics statement": Given the vulnerable status of asylum seekers, did the authors make sure that their research did not fall under a CPP, according to the Jardé Law as revised in 2016? | Thank you very much for this comment because the ethics issue was central in performing this study.  As stated in the letter provided to the Editorial office of the journal as supporting documentation for our submission, the CPP of Dijon confirmed that this study did not require ethics committee approval.  We have also specified this in the revised version, for greater clarity. |
| Regarding "Data Availability": The authors indicate that there are restrictions on data accessibility but do not specify which ones. | Thank you for bringing this omission to our notice. We have added the relevant information in the manuscript. |
| Regarding the COREQs: I suggest indicating directly information in the table, rather than indicating where in the text it can be found. | Thank you, the COREQ has been modified as requested. |
| Regarding Vancouver editing reference style: In the text, please use [], not (). | Thank you for pointing this out – we have made the appropriate corrections to the reference style. |

Journal Requirements:

When submitting your revision, we need you to address these additional requirements.

1. Please ensure that your manuscript meets PLOS ONE's style requirements, including those for file naming. The PLOS ONE style templates can be found at

https://journals.plos.org/plosone/s/file?id=wjVg/PLOSOne_formatting_sample_main_body.pdf and

<https://journals.plos.org/plosone/s/file?id=ba62/PLOSOne_formatting_sample_title_authors_affiliations.pdf>

***> Our manuscript now meets PLOS ONE’s style requirement.***

2. In your Data Availability statement, you have not specified where the minimal data set underlying the results described in your manuscript can be found. PLOS defines a study's minimal data set as the underlying data used to reach the conclusions drawn in the manuscript and any additional data required to replicate the reported study findings in their entirety. All PLOS journals require that the minimal data set be made fully available. For more information about our data policy, please see http://journals.plos.org/plosone/s/data-availability.

Upon re-submitting your revised manuscript, please upload your study’s minimal underlying data set as either Supporting Information files or to a stable, public repository and include the relevant URLs, DOIs, or accession numbers within your revised cover letter. For a list of acceptable repositories, please see http://journals.plos.org/plosone/s/data-availability#loc-recommended-repositories. Any potentially identifying patient information must be fully anonymized.

Important: If there are ethical or legal restrictions to sharing your data publicly, please explain these restrictions in detail. Please see our guidelines for more information on what we consider unacceptable restrictions to publicly sharing data: http://journals.plos.org/plosone/s/data-availability#loc-unacceptable-data-access-restrictions. Note that it is not acceptable for the authors to be the sole named individuals responsible for ensuring data access.

We will update your Data Availability statement to reflect the information you provide in your cover letter.

***> Data Availability statement has been modified as requested.***

3. Please include captions for your Supporting Information files at the end of your manuscript, and update any in-text citations to match accordingly. Please see our Supporting Information guidelines for more information: <http://journals.plos.org/plosone/s/supporting-information>.

**> *Captions have been added as requested.***
