# Supplementary material for: Perception of the use of a telephone interpreting service during primary care consultations: A qualitative study with allophone migrants
Source: PLoS One. 2022 Mar 15;17(3):e0264832. doi: 10.1371/journal.pone.0264832 (PMC8923434; doi:10.1371/journal.pone.0264832)
Supplement: Attachment — Submitted filename: Response to Reviewers.docx [file pone.0264832.s004.docx]

**PONE-D-21-18663**

Revisions for submission: PONE-D-21-18663 ‘*Perception of the use of a telephone interpreting service during primary care consultations: A qualitative study with allophone migrants’*

Thank you very much for your feedback on our work. Please find our responses to the reviewers’ comments in the following table.

| **Review 05022022 – Minor Review** | |
| --- | --- |
| **Reviewers Comment** | **Authors Response** |
| **Reviewer 2** | |
| Thank you to the authors for responding to my comments and the revisions they have made. The manuscript has gained in quality, and I have enjoyed reading this new version. I have a few more, minor comments or questions. | Thank you for your positive appreciation of our modifications. |
| I came across a recent article on TIS over the Christmas vacation, which I believe would back up even more the introduction. It is not referenced in PsycInfo or PubMed, which is why -I’m guessing- neither the authors nor I have seen it before. It is, however, accessible on Researchgate upon request.  --> René de Cotret, F., Beaudoin-Julien, A.-A., & Leanza, Y. (2020). Implementing and managing remote public service interpreting in response to COVID-19 and other challenges of globalization. Meta LXV, 3. | Thank you very much for bringing this excellent paper to our notice. Prof. Leanza kindly sent it to us. This paper is highly relevant as it proposes guidelines for implementing videoconference-based remote interpreting services. We have referred to it in the discussion section. |
| Two other articles from Leanza’s research team also seem relevant for the discussion.  --> For the relational dynamic in interpreted consultation, including issue of trust (cf. lines 262-265) : Brisset, C., Leanza, Y. & Laforest, K. (2013). Working with interpreters in health care: A systematic review and meta-ethnography of qualitative studies. Patient Education and Counseling, 91, 131-140.  --> A study conducted in primary care (cf. lines 312-314): Brisset, C., Leanza, Y., Rosenberg, E. et al. (2014). Language barriers in mental health care: A survey of primary care practitioners. Journal of immigrant and minority health, 16(6), 1238-1246. | Again, we thank the Reviewer for bringing these publications to our notice. They are indeed of great interest and this team has performed excellent work on this topic.  We have cited these two references in the introduction section, since the sentence cited by the Reviewer as being relevant is actually in our results section, where it is not customary to include references.  The second publication is a study that investigates the point of view of primary care mental health practitioners, whereas our study presents the experience of the patients, which has been lacking heretofore in the literature. |
| * Line 44: The authors indicate two themes, while presenting three in the result section. | Thank you for your attentiveness. We have corrected this error. |
| * Lines 85-86: The authors indicate that interpreters make more strategic additions during TIS. Although I find this results a surprising (and I will not argue on it), the complete sentence seems to me shaky. Strategic additions are a positive thing while the beginning of the sentence (and the following one) emphasizes on negative aspect of TIS. | The studies we cite discuss that the number of strategic additions increases on the telephone compared to face-to-face. We replaced "and" with "while" to minimize the negative aspect of our sentence. |
| * Lines 90-93: Would the authors consider moving the connector “Nevertheless” to the beginning of the next sentence? | Of course, thank you. This does improve the flow of the sentences. We have corrected accordingly. |
| * Lines 110-113: I wonder whether it is relevant (or not) to mention that the authors have followed the IMRAD structure, as the conducted research is prospective study. | The IMRAD structure is a convention for structuring scientific articles and it is the standard structure applied for all Plos One manuscripts. Therefore, we do not believe it is necessary to specify that here. |
| * Lines 135-149: I first wondered here if the authors were able to ensure that it was a different interpreter than the one(s) used during the TIS consultations, which led me to wonder if patients using TIS are actually informed of who the person interpreting for them is… | To the best of our knowledge, the patients using TIS are not informed of who the person interpreting for them is. They are simply put in contact with a person who speaks their mother tongue, and who introduces themselves as being the interpreter. |
| * Line 184: Is it pertinent to add the %? I know it is a statistical standard but the sample relies on 10 participants… | Thank you for pointing out this very relevant point. We have removed the percentages. |
| * Lines 199-200: For table 2, would the authors consider presenting a figure instead of a table? I also found two typos: a lower-case s at “subclusters” instead of a capital S; and there is a lost ”;” at the end of “the lack of an image” | We did originally consider presenting a figure instead of a table but in the end, we could not find a suitable format that would portray the information as conveniently as a table. Therefore, with all due respect, we have chosen to retain the table.  We have corrected the typos you kindly brought to our notice. |
| * Lines 215-217: Would the authors considering making two sentences here? | Thank you for this suggestion, which makes the text easier to read. We have divided it into two sentences. |
| * Line 231: I would change “physician” for “doctor” to be consistent with the rest of the paper. | We have replaced “physician” by “doctor” as suggested. |
| * Lines 257-206: Striking result which emphasizes the need to inform asylum seekers of their rights… | Thank you very much. This is why we firmly believe that research among allophone migrants is necessary and useful, and deserves to be pursued. |
| * Line 286: I would change the title of the sub-heading to “advantage and limitations of the TIS” as this section is not only about limits. | Thank you very much.  We have changed the subheading title as suggested. |
